# Negative illness feedbacks: High‐frisk policing reduces civilian reliance on ED services

**DOI:** 10.1111/1475-6773.13554

**Published:** 2020-09-25

**Authors:** Erin M. Kerrison, Alyasah A. Sewell

**Affiliations:** ^1^ School of Social Welfare University of California, Berkeley Berkeley California; ^2^ Department of Sociology Emory University Atlanta Georgia

**Keywords:** health care, help‐seeking, hospital, legal epidemiology, policing, trauma centers, utilization

## Abstract

**Objective:**

This paper demonstrates that localized and chronic stop‐question‐and‐frisk (SQF) practices are associated with community members’ utilization of emergency department (ED) resources. To explain this relationship, we explore the empirical applicability of a legal epidemiological framework, or the study of legal institutional influences on the distribution of disease and injury.

**Data and Study Design:**

Analyses are derived from merging data from the Philadelphia Vehicle and Pedestrians Investigation, the National Historical Geographic Information System, and the Southeastern Philadelphia Community Health database to zip code identifiers common to all datasets. Weighted multilevel negative binomial regressions measure the influence that local SQF practices have on ED use for this population. Analytic methods incorporate patient demographic covariates including household size, health insurance status, and having a doctor as a usual source of care.

**Principal Findings:**

Findings reveal that both tract‐level frisking and poor health are linked to more frequent use of hospital EDs, per respondent report. Despite their health care needs, however, reporting poor/fair health status is associated with a substantial decrease in the rate of emergency department visits as neighborhood frisk concentration increases (IRR = 0.923; 95% CI: 0.891, 0.957). Moreover, more sickly people in high‐frisk neighborhoods live in tracts that have greater racial disparities in frisking—a pattern that accounts for the moderating role of neighborhood frisking in sick people's usage of the emergency room.

**Conclusions:**

Findings indicating the robust association reported above interrogate the chronic incompatibility of local health and human service system aims. The study also provides an interdisciplinary theoretical lens through which stakeholders can make sense of these challenges and their implications.

**What is Known on This Topic**

- Police violence is an unyielding public health crisis in ethnoracially marginalized and economically vulnerable American communities.
- Direct contact with police officers is associated with a number of poor health outcomes, including psychological distress, substance misuse and co‐morbidity, infectious disease transmission, and death.
- Indirect contact with police officers, via residence in a neighborhood with higher concentrations of proactive and lethal policing, is associated with poor health outcomes as well (negative evaluations of health, diabetes, hypertension, acute asthma, obesity, and psychological distress)—especially, among men living in aggressively surveilled areas and women living in lethally surveilled areas.

**What This Study Adds**

- An empirical application of legal epidemiology, which examines the chronic incompatibility of local health, safety, and human service system aims.
- Evidence that sicker people who live in neighborhoods with high stop‐and‐frisk rates report less frequent use of the hospital ED, while ED utilization among healthier people is unrelated to their residential exposure to frisking. Agencies tasked with fostering a safe environment appear to engage practices that curtail the use of services by those facing serious health challenges.

## 1 INTRODUCTION

Police violence is an unyielding public health crisis in a number of marginalized and vulnerable American communities.^1^, ^2^, ^3^, ^4^, ^5^, ^6^ Research on spillovers of the criminal legal system indicates that direct contact with police officers is associated with poor health outcomes including psychological distress,^7^, ^8^, ^9^, ^10^, ^11^ compounded illness conditions,^12^, ^13^ infectious disease transmission,^14^, ^15^ and death.^16^, ^17^ Even indirect police contact poses adverse health risks for those embedded in networks that endure aggressive policing practices^18^, ^19^, ^20^, ^21^, ^22^, ^23^, ^24^, ^25^, ^26^ especially, among men living in aggressively surveilled areas and women living in lethally surveilled areas.^27^ Police officers have the latitude to initiate referrals to health care, suggest the type of institution best equipped to provide care, and determine whether transfers are executed voluntarily or coercively.^28^, ^29^ Less, however, is known about the association between embeddedness in intrusive police prevalence and surveilled community members’ willingness to rely on hospital emergency room care. Thus, we consider connectivity to intrusive policing as it correlates with visits to hospital emergency departments (EDs).

Emergency room admission protocols open opportunities for legal surveillance.^30^, ^31^ For example, Leibschutz et al^26^ shared that when Black patients in an ED learned that police could and were questioning individuals arriving in municipal ambulances, their suspicion of collusion between law enforcement and health care providers peaked. Furthermore, Lara‐Millán^25^ illustrates that even when the urban poor covertly seek help for health problems, law enforcement has developed strategies to identify the location of the facilities they frequent. Officers stationed at Lara‐Millán's field site, routinely worked toward “systematically thinning the front room wait list” (2014:877) of patients whose activity did not justify criminal legal system intervention. When police conduct unsanctioned background checks of waiting room attendees, ED utilization within these surveilled communities poses an imminent threat. These sorts of institutional practices, which are sustained by police officers and accepted by hospital staff, can deter civilians from seeking much‐needed help through ED care. This study measures how localized police activity shapes such hospital utilization.

Legal epidemiology is a framework that could enhance our theoretical understanding of how collusion between law enforcement and health services influences patients’ decisions to rely on ED care. Legal epidemiology posits that law and legal practices shape health outcomes.^32^, ^33^, ^34^ Simultaneously, research has emerged focusing on how racialized policing in racialized contexts exacerbates health problems.^1^, ^4^, ^22^, ^23^, ^35^, ^36^, ^37^ While the current study was not designed to test the theoretical constructs of legal epidemiology, we examine its applicability for interpreting a patient‐centered and spatially defined relationship between state‐operated local policing practices and hospital utilization for an ethnoracially diverse urban sample. Brayne's^38^ conceptualization of “system avoidance” holds that justice‐impacted individuals avoid surveilling institutions due to their maintenance of formal, systematized, and identifiable records. Similarly, we expect to observe a relationship between systemic place‐based police intrusion and depressed reliance on hospital EDs, including those that facilitate access to trauma services.

The current study will test whether police officers’ stop‐question‐and‐frisk (SQF) practices dampen ED visitation. This association is an indicator of *negative illness feedbacks* where police tasked with fostering a safe environment engage practices that curtail the procurement of services for those facing health challenges. Such countervailing relationships produce new forms of systemic violence that reveal the systematic marginalization that governs the lives of the inequitably surveilled. In such communities, police behaviors weaken, and even disentangle, the relationship between self‐rated health and hospitalization. We account for within and between neighborhood variation in known sociodemographic factors associated with our measures of interest.

## 2 METHODS

### 2.1 Study design

This study is a cross‐sectional observational study, describing the rate of ED visits due to residential exposure to frisk (ie, a bodily pat down). The study focuses on the 18‐65 population of the Philadelphia metropolitan division (Bucks, Chester, Delaware, Montgomery, and Philadelphia Counties, PA) in the years surrounding 2015. Data on individual reports of health collected in 2014‐2015, police stop characteristics in 2015 and 2016, and neighborhood demographic characteristics in 2011‐2015 are linked by geographical identifiers for zip code provided in all three datasets.

### 2.2 Data sources

The individual data source for this study comes from the Southeastern Pennsylvania Household Health Survey (SEPA HHS), a repeated cross‐sectional telephone survey of more than 10 000 households. SEPA HHS is one of the longest‐running household health surveys in the United States and is collected regularly at approximately biennial intervals. This study uses data from 2014 to 2015, the most recent period of data collection that has been publicly released. The data are proprietary to the Public Health Management Corporation (PHMC)’s Community Health Data Base (CHDB), which serves the information needs of health and human service agencies in the Southeastern Pennsylvania region. Information is collected about residents’ health status, use of health services, and access to care. Individual respondents lived in 44 zip codes.

The contextual data for this study come from two sources. First, the City of Philadelphia's Vehicle and Pedestrian Investigation dataset (PVPI) dataset was downloaded from OpenDataPhilly (www.opendataphilly.org) on April 4, 2017. This data source provides x/y coordinates for all investigations pursued by the Philadelphia Police Department (PPD). Since the first day of 2014, data on police investigations of pedestrians and vehicles have been uploaded daily made available to the public through OpenDataPhilly by the PPD with a 14‐day lag for operational approval processes. The information compiled by the PPD includes details related to the location of the investigation, type of investigation, the race, gender, and age of the person stopped, the behaviors of the police officer during the investigation (ie, conducted a frisk, a search, or an arrest), and the discovery of any contraband due to the investigation. We focus on 830 077 investigations recorded by officers that occurred in either 2014 or 2015. All investigations were successfully geocoded to zip codes. From poststop information recorded after the stop was completed, we created a contextual dataset where data were aggregated to one of 52 zip codes where the PPD conducted investigations.

Second, we export socioeconomic characteristics from the 2015 American Community Survey (ACS): 5‐Year Data [2011‐2015] through the National Health Geographic Information Survey.^39^ Forty‐four zip code tabulation areas with shared zip code identifiers were retained for analysis.

### 2.3 Measures

The outcome of interest is the number of ED visits in the past year. Respondents were asked: “How many visits, if any, did you have to an emergency room during the past twelve months, that is, since (date one year ago) 2013/2014?” This is reported to SEPA HHS by the respondent as a count variable characterizing the number of times in the past year that the respondent has visited the emergency room (ED) for any reason. Higher values indicate more ED utilization.

The individual‐level independent variable is a five‐category self‐rated health, an ordinal variable with Likert response options (1 = Excellent; 2 = Very Good; 3 = Good; 4 = Fair; and 5 = Poor). Self‐rated health is also measured as a dichotomous indicator, where 1 = Poor/Fair and 0 = Excellent/Very Good/Good. Higher values of both outcomes indicate worse health. Self‐rated health captures lay constructions of illness and is a well‐known strong predictor of mortality.^40^

The contextual independent variable is neighborhood frisk concentration and is measured at the zip code‐level. This continuous indicator characterizes the percentage of stops in a neighborhood where police officers engage in frisking—that is, patting down a pedestrian or a driver. Unlike prior research on the illness risks of policing pedestrians,^20^, ^22^, ^23^ we evaluate data that include stops of both pedestrian and vehicular stops, instead of focusing only on pedestrians. As such, our data capture a more robust measure of frisking than prior research. To our knowledge, this is the first study that evaluates the health care costs of frisking drivers, even while much of the research on inequalities in police surveillance is built for research on drivers.^41^, ^42^, ^43^ Higher values indicate more exposure to frisking by police for people living in an area. Zip code frisk concentration is also treated as an effect modifier in the analysis.

The analysis adjusts for several individual‐level confounders of the estimated relationships among poor self‐rated health, zip code frisk concentration, and past‐year ED utilization behavior, including ethnoracial status, gender, age, household income, family poverty, total number of kids in the household, respondent work status, respondent education, marital status, health insurance status, and regular source of medical care. Age and household income are continuous variables. Age is centered at 18. Total number of kids in the household is a count variable characterizing the number of children under the age of 18 are related to the respondent by blood, marriage, or adoption, including students under the age of 18 who are living away from home and related to the respondent.

Household income includes all sources of income from anyone living at the address who is related to the respondent by blood, marriage, or adoption and is transformed so that a one‐unit change is equivalent to $10 000. Respondents are provided 26 categories from which to choose, and these categories are midpoint recoded (see Appendix S1 for values). Since there is a substantial amount of missing data on income (*n_i_* = 654. 21.3 percent; Table 1), mean substitution replacement is used to retain respondents with missing information on household income in the analytical sample, where all respondents with missing data are assigned the mean value of the nonmissing data. We also include an indicator of missingness on household income (0 = Missing; 1 = Data Present). Furthermore, we include an imputed measure of poverty status, which is provided by PHMC to include in analysis to reduce bias due to missingness. The imputed values are coded relative to the federal poverty line (FPL) for all respondents based on the reported employment status and educational level of the main wage earner. Poverty status is coded as (1) less than 100 percent of the FPL (Reference); (2) between 100 percent and 150 percent of the FPL; or (3) greater than 150 percent of the FPL.

**TABLE 1:** Descriptive statistics for full sample and by self‐rated health status: 2014‐2015 Southeastern Pennsylvania Household Health Survey (*n_ij_* = 2920) Nested in 2015‐2016 Philadelphia Pedestrian and Vehicular Dataset and 2011‐2015 American Community Survey (*n_j_* = 44)

	Full sample		E/VG/G	P/F
	Mean	SD	%	%
Outcome of interest				
# of Visits to Emergency Room	0.69	1.49	0.46	1.49***
Zip code‐level determinants of health				
% Stops with a Frisk	8.49	3.96	8.28	9.22***
% of HH with College Degree	24.49	17.05	25.81	19.93***
% of Family Income < FPL	24.79	11.53	23.8	28.21***
Individual‐level determinants of health				
Ethnoracial status				
Black Non‐Latino	40.79		38.57	48.41***
Latino	4.76		4.42	5.92
Asian Non‐Latino	1.82		2.12	0.76*
Other Non‐Latino	8.01		7.03	11.38***
White Non‐Latino (Ref.)	44.62		47.85	33.54***
Gender				
Female gendered (Ref.)	63.15		62.05	66.91*
Male gendered	36.85		37.95	33.08*
Age of respondent	46.87	11.58	45.54	50.36***
Household income				
Midpoint income recode ($)	66 253.89	60 136.29	75 417.33	33 315.55***
Missing income information	19.28		18.44	22.15*
Has income data	80.72		81.56	77.85*
Ratio of family income to FPL				
Less than 100% (Ref.)	20.1		14.51	39.3***
Between 100% and 150%	11.44		10.31	15.33***
Greater than 150%	68.46		75.19	45.37***
Respondent work status				
Non‐full time	32.33		25.74	54.93***
Unemployed	9.72		9.64	10.02
Full time (Ref.)	49.93		57.76	23.07***
Respondent education				
Less than HS Degree (Ref.)	7.5		4.87	16.54***
HS graduate	31.81		29.01	41.43***
Technical/Trade School	3.12		3.14	3.03
Some college experience	22.02		21.98	22.15
College graduate	20.38		22.87	11.84***
Postgraduate Schooling	15.17		18.13	5.01***
Total # of kids in household	0.89	1.4	0.94	0.74**
Respondent marital status				
Married/Cohabitating (Ref.)	75.07		77.27	67.53***
Widowed, separated, divorced	19.45		17.65	25.64***
Other marital status	1.78		1.81	1.67
Single	3.4		3.27	5.16*
Health care insurance				
Insured	92.43		92.53	92.11
Uninsured (Ref.)	7.57		7.47	7.89
Regular Source of Medical Care				
Yes	89.45		88.85	91.5
No (Ref.)	10.55		11.15	8.5
Chronic health conditions				
Zero	23.66		27.11	11.84***
One	46.54		49.18	37.48***
Two	28.79		23.71	50.68***
N	2920		2261	659

Note

Percentages shown for means of nominal covariates; otherwise, raw values shown. Self‐rated health status indicated by: E = Excellent, VG = Very Good, G = Good, F = Fair, and P = Poor. FPL is established based on the 2014‐2015 Federal Poverty Line. Chronic health conditions are a count of medically diagnosed obesity and hypertension. Distributions may not up the 100% due to rounding.

* *P* < .05, **P < .01, ***P < .001 (two‐tailed test for significance of *t* test or probability tests comparing average values for respondents based on designated health status).

Gender, health insurance, regular source of medical care, and chronic health conditions are dichotomous variables. Gender is characterized as male (1) or female (0). A person is indicated as having health care insurance (1) if they indicated they received health insurance through work, school, or a union; that they bought health insurance directly and paid for it in total by themselves or their family regardless of whether they did so with government assistance; that they had access to health insurance through a government program; or that they obtained health insurance through some other insurance, group, or place not including those already mentioned. All others are coded as uninsured (0). Regular source of medical care is captured by the question: “Is there one person or place you USUALLY go to when you are sick or want advice about your health?” Individuals affirming this question are coded as “yes” (1); all others are coded as “no” (0). The presence of chronic health conditions is indicated by having doctor‐diagnosed asthma, diabetes, hypertension/high blood pressure, and obesity.

Ethnoracial status, family income, respondent work status, respondent education, and marital status are measured as nominal indicators. Ethnoracial status captures respondents who self‐identified as Black non‐Latino, Latino, Asian non‐Latino, Other non‐Latino, or White non‐Latino (Reference) are included in the analysis. Respondent work status references the current employment situation and is coded as either: (1) nonfull time (including employed part‐time, retired, disabled, homemaker, student, and job training); (2) unemployed; or (3) full time (Reference). Respondent education is coded as the last grade of school that was completed and is coded as either: (1) less than high school graduate (0 to 100 years; Reference); (2) high school graduate (12 years or GED certificate); (3) technical, trade, or vocational school after high school; (4) some college but no four‐year degree or Associate's degree; and (5) college graduate or four‐year degree; (6) postgraduate or professional schooling after college. Respondent marital status is coded as (1) married or living with a partner (Reference); (2) widowed, separated, and divorced; (3) other marital status; or (4) single. Table 1 indicates the reference category (0) for dichotomous and nominal covariates.

Neighborhood‐level confounders derived from the 2015 ACS dataset account for covariance related to education and poverty status. Neighborhood educational attainment is measured by the percentage of the population over the age of 25 who has at least a college degree. Neighborhood poverty status is measured as the percentage of families whose income to poverty level ratio is less than one (1). A moderate negative correlation exists between neighborhood education and neighborhood poverty (*r* = −.59), and a moderate correlation exists between neighborhood frisk concentration and neighborhood socioeconomic status (*r*_education._ *_j_* = −.49; *r*_poverty_ *_.j_* = .53).

### 2.4 Sample

The study sample focused on people between 18 and 65 at the time of the survey administration, nested in neighborhoods surveilled by local police. Of the 9044 respondents sampled in 2014‐2015 by SEPA HHS, only 7,024 respondents (77.7 percent) met the age criteria for the study. While all SEPA HHS respondents were geocoded to a zip code by PHMC, not all these respondents lived in neighborhoods surveilled by PPD. Only 44 of the 143 geocoded zip codes have policing information (30.8 percent). Of those that meet the age criteria for this study, only 3073 respondents (43.8 percent) reside in neighborhoods where information on police behavior is available from the PPVD. The vast majority of respondents lived in Philadelphia County (*n_i_* = 3006; 97.4 percent); the remaining respondents lived in either Delaware (*n_i_* = 2) or Montgomery (*n_i_* = 65) counties.

The analytical sample, meanwhile, only retained respondents with valid data on the outcome of interest, the effect modifier, and sociodemographic and neighborhood controls. Due to the substantial proportion of cases missing data on household income (*n_i_* = 654, 21.2 percent), we employed two missing data techniques. First, we engaged in dummy‐variable replacement, including an indicator of all cases with missing household income data. Second, to reduce bias in our estimates, we included a measure imputing family poverty status, which is based on work status and educational attainment characteristics. Among the analytical sample, there is no statistically significant difference in the distributions of missing household income data across levels of family poverty (Pearson's Chi^2^(2) = 3.96; *P* = .138).

Aside from household income, listwise deletion was conducted since no individual‐level covariate contributed more than 1 percent to missing data. Only 126 (4.1 percent) of the study sample were removed from the analysis due to invalid nonincome data. Among the study sample, there is no statistically significant difference in the distributions of missing nonincome data across levels of family poverty (Pearson Chi^2^(2) = 0.29; *P* = .866). Our final sample size is 2920 in 44 neighborhoods (84.6 percent of eligible zip codes).

### 2.5 Analytic strategy

The statistical modeling framework employed in this study anticipates that individual reports of ED visits are partly a function of the respondent's health status and partly a function of the zip code in which an individual resides. There is significant clustering in the analytical sample, as there are about 66 residents on average across 44 neighborhoods. About 2.8 percent of the variance in the number of visits to the ED is attributable to zip codes. This is within the range of shared variation due to institutional attachments^44^ and the degree of clustering merits the use of multilevel models.^45^

Weighted multilevel and cross‐level negative binomial regression models with random intercepts are used to make inferences about the population of individuals living in the areas that PPD surveils. A comparable zero‐inflated negative binomial regression model was also computed in sensitivity analysis, yielding similar results. At the individual level, we use a balancing weight that accounts sample bias due to systematic patterns of nonresponse to the survey (according to race, age, sex, household size, and income characteristics of the region in the 2010 US Census), which allows us to maintain the sample size while providing analytical estimates that represent the demographics of the Philadelphia metropolitan division. At the neighborhood level, we assume that all neighborhoods surveilled by the PPD have equal likelihood of being represented in the sample. Stata 14.1^46^ is used for all analyses.

Our descriptive statistics provide a comparison of sample covariates for two key groups—those with excellent/very good/good health and those with poor/fair health. *t* tests were conducted to compare the averages of continuous and count measures across groups; proportion tests are conducted to compare the averages of dichotomous measures across groups; and chi‐square statistics are computed to compare the distributions of nominal measures across groups. We expect that respondents who report worse (poor or fair) health will live in neighborhoods (zip codes) where police are more likely to frisk pedestrians and drivers (Hypothesis 1).

We conduct three sets of regression analyses to evaluate the role of illness and neighborhood frisk exposure on ED utilization. The first regression analysis (Model 1) evaluates the past‐year rate of ED visits to self‐rated health. We expect that respondents with worse self‐rated health will utilize the ED more in the 12 months prior to participating in the SEPA HHS survey (Hypothesis 2).

The second regression analysis (Model 2) evaluates whether the rate of ED visits associated with worse self‐rated health is independent of neighborhood frisk concentration. We expect that, among those respondents with the same reported health profile, living in zip codes where police are more likely to frisk during stops will be associated with an increased rate of visiting the ED (Hypothesis 3). Essentially, self‐rated health and neighborhood frisking likelihoods are separate sources of variation in ED utilization behavior.

The third regression analysis (Model 3) evaluates an effect modifier—that is, whether the number of visits to the ED in the past year is associated with exposure to police frisking behavior in the neighborhood is different according to lay reports of health. We expect that respondents of worse health who live in zip codes where police engage more frequently in frisking more often will be less likely to visit the ED (Hypothesis 4). Confirmation of Hypothesis 4 would evidence that neighborhood frisk concentration represses, or buffers against, ED utilization.

Incidence rate ratios are shown, and 95% confidence intervals are provided in brackets. Estimates shown above one (1) indicate higher rates of ED visits, while estimates below one indicate lower rates of ED visits. Estimates of control covariates are omitted from tabulated results but are available upon request.

## 3 RESULTS

Table 1 indicates that the respondents in the sample are healthy, where only 22.6 percent of them report “poor” or “fair” health statuses. Yet, Panel A of Table 2 evaluates the rate of ED visits for those reporting better self‐rated health. The first column of estimates (Model 1) indicates that people who report better health visit the ED less in the past year (IRR = 0.732; 95% CI: 0.656, 0.816; *P* < .001). Consequently, if a person were to decrease their health status by one‐unit (eg, from “good” to “fair”), their rate of ED visits would be expected to increase by a factor of 1.367, while holding all other variables in the model constant. Hypothesis 2 is supported: People with worse health utilize the ED more often.

**TABLE 2:** Regression of frisking concentration and health status on the number of visits to the ED in Past Year: 2014‐2015 Southeastern Pennsylvania Household Health Survey (*n_ij_* = 2920) Nested in 2015‐2016 Philadelphia Pedestrian and Vehicular Dataset and 2011‐2015 American Community Survey (*n_j_* = 44)

	Model 1	Model 2	Model 3
Panel A			
Better SRH	0.732*** [0.656, 0.817]	0.734*** [0.658, 0.819]	0.593*** [0.464, 0.758]
ZC Frisk Concentration		0.973 [0.942, 1.006]	0.925** [0.8468, 0.964]
Better SRH X Frisk Concentration			1.024* [1.004, 1.045]
Panel B			
Excellent/Very Good Health	0.403*** [0.301, 0.541]	0.407*** [0.303, 0.547]	0.201*** [0.109, 0.366]
Good Health	0.639*** [0.517, 0.789]	0.641*** [0.517, 0.795]	0.329*** [0.204, 0.533]
Neighborhood Frisk Concentration		0.975 [0.944, 1.008]	0.923*** [0.891, 0.957]
E/VG X Frisk Concentration			1.083** [1.031, 1.139]
G X Frisk Concentration			1.076*** [1.029, 1.124]

Note

Neighborhood frisk concentration in Panel B is evaluated when the nominal self‐rated health measure is equal to the reference category. The reference category for Panel B is Poor/Fair Health. Exponentiated coefficients shown; 95% confidence intervals in brackets. All models adjust for sociodemographic attributes, health care characteristics, and neighborhood socioeconomic status.

Abbreviations: E, Excellent; F, Fair; G, Good; P, Poor; SRH, Self‐Rated Health; VG, Very Good.

* *P* < .05, **P < .01, ***P < .001 (two‐tailed test for statistical significance).

Model 2 includes a covariate for zip code‐level frisk concentration to examine whether living in areas where police are more likely to pat down people is associated with the rate of ED visits. Zip code frisk concentration is not statistically significant. Supplemental analyses indicate that similar patterns are evident when not controlling for lay health. There is no association between area‐level pat downs and ED visits in Philadelphia. Hypothesis 3 is not supported—among people with the same health profile, living in areas where police are more likely to engage in frisking does not distinguish their ED utilization.

Model 3 includes an interaction term between better self‐rated health and zip code frisk concentration; this regression analysis tests whether the number of ED visits for people in better health is buffered or amplified by living in areas where police pat down people more. Better health continues to deter ED utilization. Nonetheless, the main association of living in areas with high levels of surveillance via frisking is statistically significant, as is the interaction term denoting effect modification to the health care costs of police frisking. Holding all other covariates in the model constant, each percentage increase in areal exposure to police frisking is expected to decrease the rate of ED visits by a factor of 0.925 significant (IRR = 0.925; 95% CI: 0.847, 0.964; *P* < .001). Reporting better health, however, negates the rate of ED visits associated with residential exposure to frisking (IRR = 1.024; 95% CI: 1.003, 1.045; *P* < .05). Healthfulness buffers against the rate of ED use that is associated with the prevalence of police frisking pedestrians—Hypothesis 4 is supported.

Panel B of Table 2 employs a nonlinear functional form for self‐rated health that distinguishes the rate of ED visits for two status groups—those reporting “poor” or “fair” health and those reporting “excellent,” “very good,” or “good” health. Models 1‐3 of Panel B yield similar estimates as Panel A; however, a more concrete interpretation of the moderated association of area‐level frisk concentration emerges. For people of poor/fair health (ref.), there is a substantial decrease in the rate of ED visits as neighborhood frisk concentration increases (IRR = 0.923; 95% CI: 0.891, 0.957; *P* < .001). Meanwhile, ED utilization for healthier people is not affected by residential exposure to frisking. For instance, after taking into consideration the interaction term assessing the relative rate of ED utilization associated with exposure to police frisking for those in excellent/very good health compared to those in poor/fair health (IRR = 1.083; 95% CI: 1.031, 1.139; *P = *.002), testing linear combination of parameters reveals there is effectively no change in the rate of ED visits among the healthiest population (IRR = 1.001; 95% CI: 0.956, 1.046; *P* = .996). Supplemental analysis reveals similar patterns for those who report “good” health.

**FIGURE 1:**
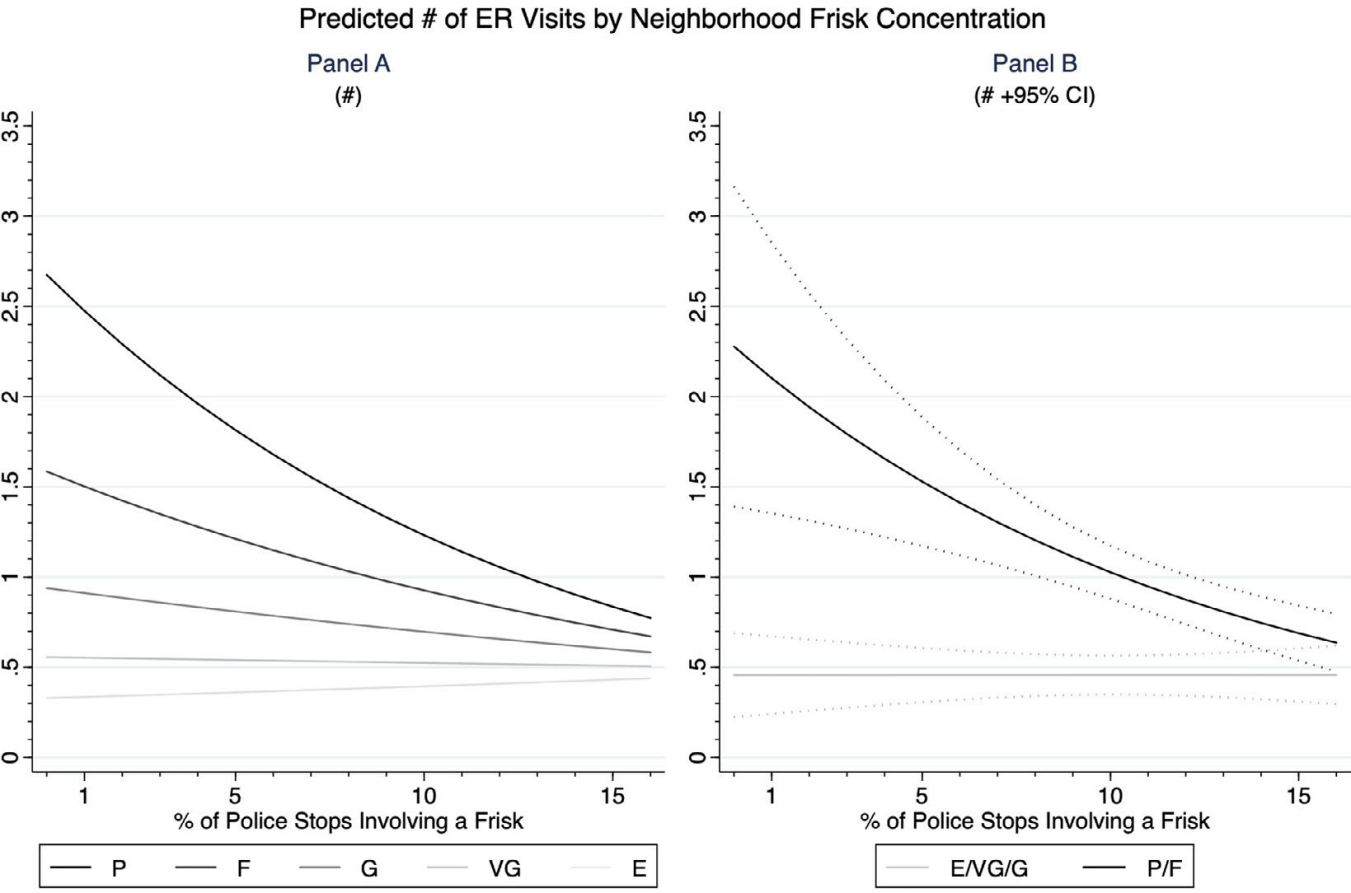
Predicted # of ER visits by neighborhood frisk concentration. E, Excellent; F, Fair; G, Good; P, Poor; VG, Very Good

Figure 1 illustrates buffering processes using simulation techniques that predict the number of ED visits in the past 12 months. Panel A shows the moderated associations of a linear measure of lay reports of health, while Panel B shows the moderated associations of the nominal measure of self‐rated health and depicts its nonlinear functional form. To demonstrate the substantial and statistically significant rate of ED visits associated with living in areas with higher exposure to police frisking, Panel B of the figure provides confidence intervals for the predicted number of visits for people in the poor/fair and excellent/very good health categories. At the lowest levels of neighborhood frisking, sick people visit the ED over 2 times a year, while this same group of people visit the ED less than 1 time a year at the highest levels of neighborhood frisking. Meanwhile, healthy people visit the ED less than 1 time a year for the full range of values. The number of ED visits for the sick and the healthy converges in neighborhoods where police conduct frisking in 14 percent of stops, a value that is roughly equivalent to the 19th percentile of this covariate. The negative rate of change in the numbers of visits for sick people coupled with the flat rate of change in the number of visits for healthy people provides evidence of a strong buffering relationship, where police frisking practices in the neighborhood repress ED visits for the sickest residents of the city. Additional study results are detailed in Appendix S2.

## 4 DISCUSSION

Ostensibly, emergency health care providers and local law enforcement endeavor to adhere to similar missions of treating people in crisis. However, the danger that accompanies police presence and questioning in a community context already characterized by police intrusion undermines these institutions’ alleged shared goals. While we acknowledge that this study is limited by its cross‐sectional design and its examination of geographically based contexts that are structurally under‐resourced and underserved, we believe that these findings offer important health care provision and theoretical implications for addressing this conflict.

First, our findings suggest that racial discrimination perpetuates intergenerational and epigenetic adverse health. Thus, place‐based structural determinants of health should be incorporated more directly into patient‐centered care.^47^ Limited community access to regular health care is a pronounced risk factor for injurious self‐medication and a host of other behaviors that invite police surveillance. Simultaneously, communities marked by police disruption also maintain high unemployment rates and an aggregated disqualification from a range of social services that have direct health implications (eg, a higher prevalence of uninsured households). The legal estrangement and cynicism that policed communities transfer to other state institutions limits the quality of their citizenship and capacity to lead healthy lives.^48^, ^49^, ^50^, ^51^, ^52^

Second, hospitals must provide deidentified emergency care to all, regardless of their race or ethnicity, the location of where injury or illness originated, or their financial capacity.^53^, ^54^ Though guaranteed by US law, communities marked by chronic criminal legal system intrusion are de facto excluded from accessing public health care provision. Consistent with the assumptions of a legal epidemiological framework underscoring these relationships, policing in this study appears to indirectly set a context for exposure to injury and hastens illness by limiting access to care for the communities under its heel. In response, we hope that proponents that advocate for the right to health and health care leverage the authority of the law to equitably (re)distribute access to care and hold public health systems accountable to the promises of their charge.

We conclude by considering the ethical and future research implications of the results for the behaviors of health care professionals and providers. Our findings highlight the health care consequences of two interspersed sources—embeddedness in a legally sanctioned highly surveilled community and reliance on the medical community for access to the sick role. However, most health care administrators and professionals do not draft intake protocols in collaboration with community members who understandably fear medical intervention and engage in self‐isolating practices in the presence of and as a result of localized police presence. Our results, then, provide support for the utility of empirical inquiry into social medicine, which calls medical professions to go *into* the community instead of waiting for its residents to pass through their doors.^55^, ^56^ Process and impact evaluations could assess the merits of these processual remedies, including a dedicated publicly funded investment in regulating and supporting the viable operation of these centers, the patients who would rely on their services, and the law enforcement agents responsible for the safety of all parties moving through those sites. A legal epidemiological framework for imagining the law's potential to positively shape these health‐promoting possibilities merits scholarly and political attention.

Namely, explicit policy and protocols that limit police presence inside the emergency and trauma care setting could reduce hospital utilization disparities. We also recommend a committed consideration of alternatives to police presence that not only reduce the routine disruptions that are linked to criminal legal system intrusion, but allow for the cultivation of community‐based, community‐built, and community‐directed health and wellness promoting institutions.^57^, ^58^, ^59^ Such alternatives must engage health care professionals to remove their role in the criminalization process. Finally, we advance the belief that people know what they need. Yet, the sickest among them who know they are in need must also weigh that urgency against a reasonable desire to keep themselves and those dearest to them, alive. This calculation is not a choice that anyone should consider, let alone routinely make.

## Supporting information

Author Matrix

Appendix S1‐S2
