# Supplementary material for: Negative illness feedbacks: High‐frisk policing reduces civilian reliance on ED services
Source: Health Serv Res. 2020 Sep 25;55(Suppl 2):787–96. doi: 10.1111/1475-6773.13554 (PMC7518820; doi:10.1111/1475-6773.13554)
Supplement: Supplementary file 2 — Appendix S1‐S2 [file HESR-55-787-s002.docx]

| **APPENDIX S1. Description of Household Income Measure: 2014-2015 Southeastern Pennsylvania Household Health Survey (*n_ij_* = 2,920)** | | | | | |
| --- | --- | --- | --- | --- | --- |
| Nominal Indicator | Description of Category Range | Midpoint Recode | *N* | % of *N* | Cumulative Percent |
| 1 | Less than $5,850 | 2,925 | 101 | 3.46 | 3.46 |
| 2 | $5,850 to under $7,850 | 6,850 | 51 | 1.75 | 5.21 |
| 3 | $7,850 to under $9,900 | 8,875 | 90 | 3.08 | 8.29 |
| 4 | $9,900 to under $11,700 | 10,800 | 57 | 1.95 | 10.24 |
| 5 | $11,700 to under $14,000 | 12,850 | 50 | 1.71 | 11.95 |
| 6 | $14,000 to under $15,700 | 14,850 | 44 | 1.51 | 13.46 |
| 7 | $15,700 to under $17,500 | 16,600 | 85 | 2.91 | 16.37 |
| 8 | $17,500 to under $18,000 | 17,750 | 43 | 1.47 | 17.84 |
| 9 | $18,000 to under $20,000 | 19,000 | 62 | 2.12 | 19.97 |
| 10 | $20,000 to under $23,750 | 21,875 | 85 | 2.91 | 22.88 |
| 11 | 23,750 to under $28,000 | 25,875 | 106 | 3.63 | 26.51 |
| 12 | $28,000 to under $29,750 | 28,875 | 41 | 1.4 | 27.91 |
| 13 | $29,750 to under $31,900 | 30,825 | 70 | 2.4 | 30.31 |
| 14 | $31,900 to under $36,000 | 33,950 | 83 | 2.84 | 33.15 |
| 15 | $36,000 to under $40,000 | 38,000 | 84 | 2.88 | 36.03 |
| 16 | $40,000 to under $41,900 | 40,950 | 41 | 1.4 | 37.43 |
| 17 | $41,900 to under $47,800 | 44,850 | 103 | 3.53 | 40.96 |
| 18 | $47,800 to under $55,400 | 51,600 | 102 | 3.49 | 44.45 |
| 19 | $55,400 to under $60,000 | 57,700 | 58 | 1.99 | 46.44 |
| 20 | $60,000 to under $63,900 | 61,950 | 83 | 2.84 | 49.28 |
| 21 | $63,900 to under $72,000 | 67,950 | 122 | 4.18 | 53.46 |
| 22 | $72,000 to under $75,000 | 73,500 | 77 | 2.64 | 56.1 |
| 23 | $75,000 to under $100,000 | 87,500 | 204 | 6.99 | 63.08 |
| 24 | $100,000 to under $150,000 | 125,000 | 304 | 10.41 | 73.49 |
| 25 | $150,000 to under $250,000 | 200,000 | 142 | 4.86 | 78.36 |
| 26 | $250,000 or over | 250,000 | 69 | 2.36 | 80.72 |
| DK/REF | Missing | 86,624 | 563 | 19.28 | 100 |
|  | | | | | |

# Appendix S2—Additional Study Results Details

## Descriptive Statistics

Table 1 indicates that the mean number of visits to the ED is less than 1. However, the distribution for the outcome of interest is skewed to the left, with some respondents reporting frequent ED visits. Most of the sample are healthy: 47.3% report “excellent” or “very good” health, 30.1% report “good” health, and 22.6% report “poor” or “fair” health. On average, respondents live in neighborhoods where 8.4 percent of suspects are frisked by the police, where 24.5 percent of households hold a college degree, and 24.8 percent of families live below the federal poverty line (FPL).

The sample is predominately Black non-Latino (40.8%) and White non-Latino (44.6%) with a smattering of ethnoracial status groups: 4.8% Latino, 1.8% Asian non-Latino, and 8% Other non-Latino. The predominately female-gendered sample is on average 46.9 years old. The average household brings in $66,253 in income and lives at least 150% above the federal poverty line. Approximately half of respondents are working full time currently, and over half have at least some college experience. The average household has less than 1 child under the age of 18 living in the house. The majority of respondents report being married or living in a cohabitating relationship, having health care insurance, and having a regular source of medical care. Only a quarter of the sample report not being diagnosed hypertension or high blood pressure nor meeting the medical threshold for obesity due to gender, or medically diagnosable obesity.

There are, however, substantial differences in sample covariates based on the key health groups. People in poor/fair health have at least one additional visit to the ED in the past year than people in better health and report worse health. They also live in neighborhoods where a higher percentage of the public is subject to frisking, providing support for Hypothesis 1—that people in worse health live in neighborhoods with a higher concentration of police engaging in frisking. People in poor/fair health are more likely to be Black non-Latino and Other non-Latino and less likely to be Asian non-Latino or White non-Latino. Female-gendered persons are more likely than male gendered people to be in poor/fair health as are people who are older in age and who have access to less household and family income. People in poor/fair health are less likely than those in better health to provide income data and are more likely to not work full time (and less likely to work full time). People in worse health have completed less years of school than people in better health and have less kids in the household. While people in poor/fair health are less likely to be married/cohabitating, they are more likely than their better health counterparts to be widowed, separated, divorced, single and to have a regular source of medical care.

## Other Analysis

To address the reliability of our analysis due to our conceptualization of neighborhoods as zip codes, we evaluated neighborhood policing at a smaller unit of analysis. Specifically, we assessed the rate of ED visits associated with police frisking clustered at the level of the census tract. This sensitivity analysis indicated that there was no evidence of effect modification with census tract-level measures of police frisking concentration.

To address the reliability of our analysis due to our conceptualization of illness, we evaluate evidence of effect modification related police frisking behavior using measures of the number of chronic health conditions as operationalized in the set of controls used in the main analysis. For this sensitivity analysis, we evaluate the role that the accumulation of chronic health conditions plays in shaping the ED visits while accounting for lay reports of health by including a cross-level interaction term allowing for the fixed effect of neighborhood frisk concentration to vary by the number of chronic health conditions. We identify consistency in the rate of ED visits of neighborhood frisk concentration across the range of chronic health conditions. These results suggest that rate of ED visits operate through lay construction of illness—that is, an individual’s perception that they are ill and in need of the sick role to which ED providers can give them access.

Since our main analysis (not shown) confirms that people who have a regular source of care visit the ED more (IRR = 1.45; 95% CI: 1.064, 1.976; *p* = 0.019), we may not be measuring ED utilization behavior, per se. Rather, we may be measuring a person’s reliance on the ED as a regular source of care or the likelihood that a person lacks a usual source altogether. To evaluate this general thesis, we composed a more detailed measure of usual type of care, splitting respondents into those with no regular source of care (*n_i_* = 308; 10.55%) and those whose regular source of care is a hospital ED (*n_i_* = 60; 2.05%), a doctor’s office (*n_i_* = 1, 967; 67.36%), or some other place (*n_i_* = 1,094; 12.14%). However, sensitivity analyses indicate that *sicker* people living in highly surveilled areas are actually *more* likely to have a regular source of care (OR = 1.029; 95% CI: 1.001, 1.058; *p* = 0.041). Moreover, people living in highly surveilled areas are not more or less likely to use the hospital ED as a regular source of care (OR = 1.008; 95% CI: 0.881, 1.153; *p* = 0.904). Further analysis indicates that all persons in poorer health, regardless of the regular presence or usual source of care, experience a decreased rate of ED visits if they live in highly surveilled areas. Supplemental analysis is available upon request.

There are possible sources of confounding due to omitted variables at the neighborhood level—specifically, the ethnoracial composition of the area, the concentration of young children in the area, the employment status of the main wage earner of the household, and the modal family structure of the area. We consider these intervening variables at the neighborhood level. Moreover, due to high correlation of some of these variables with neighborhood economic characteristics, the inclusion of these covariates would challenge the validity and reliability of our estimates due to issues of multicollinearity. Finally, with only 44 neighborhoods, we are limited in the number of variables that can be introduced at the neighborhood level due to restricted degrees of freedom.

Our estimation of the rate of ED visits associated with neighborhood frisk concentration is subject to bias from at least three sources. There may be a different neighborhood distribution of frisking based on the location (on the street vs. in the car), the seasonality (cold vs. warm months), and the timing (day or night) of police stops. One could estimate neighborhood-level measures of each of these sources of bias. However, an analysis of such variables with regard to their health(care) consequences raises issues that are beyond the scope of this research. Future research should examine effect modification due to a person’s reason for ED visits and the severity of the illness condition that prompted the ED visit. This type of information was not ascertained in the PHMC CHDB.
